# Understanding Nurses' and Physicians' Knowledge, Use and Perspectives on Nitrous Oxide/Oxygen Use in Paediatrics: A Cross‐Sectional Study

**DOI:** 10.1111/nicc.70360

**Published:** 2026-02-02

**Authors:** Valentina Simonetti, Beatrice Gullo, Ilenia Stracci, Davide Miniscalco, Elisa Capriotti, Silvia Oroli, Michela Arragoni, Lamberto Manzoli, Giancarlo Cicolini, Dania Comparcini

**Affiliations:** ^1^ Department of Innovative Technologies in Medicine & Dentistry “G. D'annunzio” University of Chieti‐Pescara Chieti Italy; ^2^ Department of Biomedicine and Prevention University of Rome “Tor Vergata” Rome Italy; ^3^ Azienda Sanitaria Territoriale (AST) Ascoli Piceno Ascoli Piceno Italy; ^4^ Health House Residence Anni Azzurri Campofilone Fermo Italy; ^5^ Azienda Sanitaria Territoriale (AST) Health Territorial Services Ascoli Piceno Italy; ^6^ Department of Medical and Surgical Sciences Alma Mater Studiorum, University of Bologna Bologna Italy; ^7^ Interdisciplinary Department of Medicine University of Bari “Aldo Moro” Bari Italy

**Keywords:** conscious sedation, cross‐sectional study, inhalation analgesia, nitrous oxide, paediatric emergency medicine

## Abstract

**Background:**

Nitrous oxide/oxygen (N_2_O/O_2_ 50%/50%) is an effective and safe technique for procedural sedation in paediatric settings; however, the knowledge, use and perspectives of healthcare professionals regarding N_2_O/O_2_ remain limited.

**Aim:**

To investigate the knowledge, use and perspectives of healthcare professionals regarding N_2_O/O_2_ in maternal–infant and emergency units.

**Study Design:**

A cross‐sectional study was conducted (March to December 2024) using (i) a sociodemographic information tool and (ii) a 16‐item questionnaire assessing knowledge, clinical use, perceived barriers and willingness to adopt N_2_O/O_2_. Descriptive and multivariate analyses were performed.

**Results:**

Of 113 respondents, 40.7% reported currently using N_2_O/O_2_ in their clinical units, while 91.0% of non‐users expressed willingness to adopt it. Barriers included lack of equipment (92.9%) and training (84.1%). 91.1% considered N_2_O/O_2_ ethically acceptable, consistent with principles of beneficence, non‐maleficence and child comfort. Employment in emergency units and holding a three‐year nursing degree were significant predictors of N_2_O/O_2_ use.

**Conclusions:**

Limited Use of N_2_O/O_2_ in Italian Paediatrics Contrasts With Strong Professional Interest. Improving Training and Resources Could Enhance Access to Safe Paediatric Sedation.

**Relevance to Clinical Practice:**

Training, equipment and guidelines are needed to translate willingness into real clinical practice, improving the safety and consistency of paediatric sedation.

## Impact Statements

- What is known about the topic
  ○ Nitrous oxide/oxygen is a safe and effective method for sedation in paediatrics.
  ○ There is limited knowledge about healthcare professionals' experiences, perspectives and attitudes regarding the use of N_2_O/O_2_ in paediatric procedural sedation.
- What this paper adds
  ○ Nurses and physicians are willing to use it, but its use remains limited.
  ○ Barriers to nitrous oxide/oxygen use include a lack of protocols, equipment and training.
  ○ Setting and educational level seem to predict nitrous oxide/oxygen use in practice.

## 1 Introduction

Procedural pain is frequent in paediatric settings and often remains underestimated [1]. Conscious sedation with N_2_O/O_2_ is a well‐established technique, with proven applications in disciplines such as dentistry [2] and obstetrics [3]. Specifically, in paediatric dentistry, N_2_O/O_2_ is widely recognised as a safe behaviour management technique (BMT) [4], for its anxiolytic and mild sedative effects that help to reduce fear or anxiety, limit unwanted movements and enhance cooperation during procedures [2]. Accordingly, in paediatric practice, N_2_O/O_2_ is widely recommended as a first‐line option for cooperative children and adolescents undergoing mildly to moderately painful procedures [5]. Despite its effectiveness, little is known about its practical use and perspective in paediatrics.

## 2 Background

International literature supports the use of N_2_O/O_2_ for various minor procedures in paediatric care, including peripheral venous catheter insertion [6], lumbar punctures and intramuscular injections [7] and broader procedural analgesia [8].

Its safety has been confirmed by the absence of serious adverse events during or after sedation, in accordance with international standards of pharmacological tolerability [9]. Adverse effects associated with N_2_O/O_2_ are typically mild and self‐limiting, including dysphoria, dizziness, nausea and headache [10]. Compared to intravenous ketamine, N_2_O/O_2_ has a safer profile, fewer side effects and faster recovery while maintaining procedural efficacy [11]. Most of the existing literature has primarily focused on specific clinical settings, such as paediatric dentistry [12], oncology/haematology short stay units [13] and emergency department [14].

Although N_2_O/O_2_ is widely recognised as an effective method for its efficacy in paediatric procedural sedation [15], little is known about how N_2_O/O_2_ is applied in clinical practice, as well as how it is perceived by healthcare workers in terms of safety, effectiveness and ethical acceptability.

## 3 Aims

The aim of this study is to investigate the knowledge, use and perspectives on N_2_O/O_2_ in paediatrics among medical and nursing staff working in an Italian paediatric and emergency units.

## 4 Design and Methods

A monocentric cross‐sectional study was conducted from March to December 2024.

The Strengthening the Reporting of Observational studies in Epidemiology (STROBE) guidelines [16] were used for reporting.

### 4.1 Setting and Sampling

All physicians and nurses employed in the maternal infant and emergency units of a public healthcare organisation in Central Italy (Azienda Sanitaria Territoriale (AST) Ascoli Piceno), working full‐time or part‐time, permanent or temporary, who cared for paediatric patients aged 3–16 during the study period were enrolled, as maternal–infant units in the Italian healthcare system often include children and adolescents, with the upper age limit for paediatric care varying between 14 and 18 years, depending on the regions and hospitals. Written informed consent was obtained. Those who did not meet the criteria or declined participation were excluded.

A non‐probability convenience sampling method was employed. A sample of 100 subjects was needed to achieve a reasonably precise 95% confidence interval of +/−10% around an estimated 50% proportion of N_2_O/O_2_ use.

An 80% response rate was expected, in accordance with methodological standards [17].

### 4.2 Data Collection Tools and Methods

Before data collection began, a researcher informed Clinical Directors and Chief Nurses of the study's purpose and methods. Data collection was planned to minimise interference with clinical workflows. In each unit, the same researcher managed recruitment, providing verbal and written information about the study, confidentiality and voluntary participation.

Participants provided written informed consent, stored separately from their questionnaires to preserve anonymity. They completed a 15‐min self‐administered printed questionnaire, usually during breaks and submitted it anonymously by placing it in a sealed envelope and depositing it into a locked ‘blind box’ in the unit.

The data collection instrument was a self‐administered questionnaire composed of two main sections: (i) Sociodemographic Information, which included participants' age, sex, nationality, department, professional role, education and years of professional experience; (ii) a self‐administered 16‐item questionnaire by Alkandari et al. (2016) [12] to investigate the participants' knowledge, use and attitudes toward N_2_O/O_2_.

Questionnaire items were grouped into four domains: (i) use and inclination to use N_2_O/O_2_; (ii) knowledge about guidelines, laws, requirements and risks associated with N_2_O/O_2_ use for children's sedation; (iii) training and provision of education on N_2_O/O_2_ use; and (iv) ethical and organisational perspectives regarding N_2_O/O_2_ use.

Response mode varied according to the item type. Questions assessing usage and inclination to use, knowledge about guidelines, training, ethics and perspectives required dichotomous (yes/no) answers. Items regarding participants' perspective about frequency of N_2_O/O_2_ usage (first domain), the risks associated with the use of N_2_O/O_2_ (second domain) and the reasons why in N_2_O/O_2_ is not still widely used in paediatric settings (fourth domain) required participants to select more than one response from a list of possible answers. Also, the items on participants' perspective on N_2_O/O_2_ as a cost‐effective and safe sedation treatment were assessed using a 3‐point Likert scale (1 = agree, 2 = neutral, 3 = disagree).

The instrument followed a validation process as described below.

#### 4.2.1 Phase 1: Linguistic Validation

Between May and June 2023, following formal authorization to use the instrument by Alkandari et al. (2016) [12], a linguistic validation process was carried out using a validated forward–backward translation method [18]. Initially, two native Italian‐speaking researchers independently translated the original English version into Italian. Discrepancies were resolved through consensus with the involvement of a third researcher. The Italian version was then back‐translated into English by three native English‐speaking researchers. The back‐translated version was compared with the original to ensure semantic and conceptual equivalence.

#### 4.2.2 Content Validity

A panel of nine expert nurses from clinical and educational settings assessed the instrument's content validity using the Scale‐Level Content Validity Index (S‐CVI).

Each item was scored for *relevance* and *clarity* using a 4‐point Likert scale ranging from not relevant to very relevant and from not clear to very clear. Each item was considered relevant or clear if the experts rated it as 3 or 4 on the Likert scale (relevant/very relevant and clear/very clear). The S‐CVI was calculated by considering the average of the items' Content Validity Index (I‐CVI) [19].

All items exceeded the expected cut‐off value of ≥ 0.78. The average S‐CVI scores were 0.90 for relevance and 0.93 for clarity.

In the final phase (July 2023), the Italian version of the questionnaire was tested on 10 healthcare workers, who reported no issues or controversies.

### 4.3 Data Analysis

Univariate analyses were performed to describe the study sample and questionnaire responses. The chi‐squared test was used to initially assess differences in N_2_O/O_2_ use prevalence across potential predictors. Secondly, stepwise forward logistic regression was used to identify independent associations between N_2_O/O_2_ use and each potential determinant.

All variables were included a priori in the model, except for nationality (98.2% were Italian residents) and occupation status (nurse or medical doctor), because they were collinear with education (3‐year nursing degree; 5‐year nursing degree; degree in Medicine). Standard diagnostic procedures were adopted to check final model validity: influential observation analysis (Dbeta, change in Pearson chi‐square and similar), multicollinearity, interaction terms, Hosmer–Lemeshow test for the goodness of fit and C statistic (area under the receiving operator curve) [20]. Statistical significance was defined as a two‐sided *p*‐value < 0.05 and all analyses were conducted using Stata statistical software version 13.1 [21].

### 4.4 Ethical and Institutional Approval

This study was approved by the Territorial Ethical Committee (CET) of the Marche Region on February 15, 2024, Prot. N. 2024 8.

Additionally, written institutional permission was obtained from the institution where the research was conducted. All healthcare professionals were fully informed and participated voluntarily, providing written consent with the option to withdraw at any time. Anonymity was ensured by collecting no identifying data and storing consent forms separately from the anonymous questionnaires, which were sealed in envelopes and placed in a locked ‘blind box’ in each unit. The research was conducted in accordance with the principles of the Helsinki Declaration [22].

## 5 Results

### 5.1 Demographic Results

A total of 113 healthcare professionals were included in the study. The majority were nurses (81.4%, *n* = 92). Most participants were employed in emergency units (65.5%, *n* = 74), were aged between 30 and 39 years (33.6%, *n* = 38), held a diploma or 3‐year nursing degree (53.1%, *n* = 60) and reported less than 10 years of professional experience (35.4%, *n* = 40). Detailed sociodemographic and professional characteristics are presented in Table 1.

**TABLE 1:** Characteristics of the sample, proportion of N_2_O/O_2_ users and multivariable analysis predicting N_2_O/O_2_ use.

Variables	Overall	N_2_O/O_2_ use	*p* ^a^	OR (95% CI)	*p* ^b^
	*N* (%)	*N* (%)			
Overall sample	113 (100)	46 (40.7)	—	—	—
Age class in years			0.015		
20‐29	9 (8.0)	5 (55.6)		1 (Ref. cat.)	—
30‐39	38 (33.6)	23 (60.5)		1.56 (0.26–9.23)	0.623
40‐49	32 (28.3)	8 (25.0)		0.20 (0.02–2.19)	0.185
50‐59	23 (20.4)	6 (26.1)		0.18 (0.01–2.86)	0.223
60‐69	11 (9.7)	4 (36.4)		0.32 (0.01–9.83)	0.515
Gender, *n* (%)			0.231		
Female	37 (32.7)	18 (48.6)		1 (Ref. cat.)	—
Male	76 (67.3)	28 (36.8)		1.67 (0.56–5.03)	0.360
Department, *n* (%)			< 0.001		
Emergency unit	74 (65.5)	44 (59.5)		1 (Ref. cat.)	—
Maternal and infant unit	39 (34.5)	2 (5.1)		0.03 (0.01–0.16)	< 0.001
Professional role, *n* (%)			0.446		
Nurse	92 (81.4)	39 (42.4)		1 (Ref. cat.)	—
Medical doctor	21 (18.6)	7 (33.3)		2.36 (0.17–33.2)	0.524
Education, *n* (%)			0.025		
Diploma/3‐year nursing degree	60 (53.1)	31 (51.7)		1 (Ref. cat.)	—
Master's degree or higher (Nursing)	31 (27.4)	7 (22.6)		0.18 (0.05–0.68)	0.011
Degree in Medicine	22 (19.5)	8 (36.4)		0.73 (0.05–11.3)	0.823
Years of working, *n* (%)			0.127		
0–10	40 (35.4)	20 (50.0)		1 (Ref. cat.)	—
11–20	34 (30.1)	15 (44.1)		3.25 (0.57–18.7)	0.185
21 or more	39 (34.5)	11 (28.2)		3.35 (0.34–33.4)	0.303

Abbreviations: CI, confidence interval; OR, odds ratio.

^a^ Chi‐squared test.

^b^ Logistic regression model including 113 observations. Hosmer–Lemeshow test for the goodness of fit *p*‐value: 0.53. Area under the receiving operator curve: 0.86.

### 5.2 Findings From the Questionnaire on Knowledge, Use and Perspectives Toward N_2_O/O_2_

The complete distribution of responses for each domain is shown in Table 2.

**TABLE 2:** Answers to the Alkandari questionnaire on the attitudes toward nitrous oxide/oxygen use.

Items	Overall
	*N* (%)
(i) Use and inclination to use N_2_O/O2	
I use N_2_O/O_2_ users^a^	46 (40.7)
I use N_2_O/O_2_ to reduce children's anxiety and pain^b^	46 (40.7)
I use N_2_O/O_2_ as a BMT^b^	12 (10.6)
Frequency of N_2_O/O_2_ use: ≥ 1 per week	7 (6.2)
Frequency of N_2_O/O_2_ use: ≥ 1 per month	15 (13.3)
Frequency of N_2_O/O_2_ use: ≥ 1 per year	24 (21.2)
Frequency of N_2_O/O_2_ use: never	67 (59.3)
I would use N_2_O/O_2_ if I had a chance^c^	61 (91.0)
(ii) Knowledge about guidelines, laws, requirements and risks on N_2_O/O_2_ use for children's sedation	
I am aware of the guidelines/regulations on N_2_O/O_2_ use^b^	36 (31.9)
I am aware that N_2_O/O_2_ can be used independently by a trained nurse, once it is prescribed by a physician^b^	65 (57.5)
The risks associated with N_2_O/O_2_ use, as reported by scientific studies, are	
Dysphoria, vomiting, nausea and dizziness	38 (33.6)
Respiratory distress and disorientation	7 (6.2)
All above^d^	33 (29.2)
No side effect	35 (31.0)
(iii) Training and provision of education on N_2_O/O_2_ use	
I have attended a specific course on N_2_O/O_2_ use^b^	34 (30.1)
I never attended a course, but I would like to^e^	75 (94.9)
(iv) Ethical and organisational perspectives on N_2_O/O_2_ use	
I believe that N_2_O/O_2_ sedation for the management of pain and anxiety for paediatric patients is ethical^b^	103 (91.1)
I believe that N_2_O/O_2_ sedation is cost‐effective^f^	69 (61.1)
I believe that N_2_O/O_2_ use may slow down my work^b^	25 (22.1)
Several studies state that N_2_O/O_2_ sedation in the paediatric setting is not still widely used. In your opinion, why?^g^	
Lack of facilities/equipment	105 (92.9)
Lack of training	95 (84.1)
Lack of parent's agreement	54 (47.8)
Personnel safety	21 (18.6)
Parent's disagreement	5 (4.4)
Children's refusal	1 (0.9)
It is illegal	0 (0.0)
I believe that N_2_O/O_2_ sedation during non‐invasive paediatric treatments (application of stitches, sutures, peripheral venous catheter placement, burn dressing, etc.) is safe^f^	80 (70.8)
I think that the parents are aware of the possibility of conscious sedation using N_2_O/O_2_ for their children^b^	9 (7.9)
I think that once informed about the possibility of conscious sedation, the parents would give their consent^b^	99 (87.6)

Abbreviation: BMT, behaviour management technique.

^a^ Calculated on the basis of the frequency of N_2_O/O_2_ use.

^b^ Restricted to the participants who answered ‘YES’ regarding the use of N_2_O/O_2_ of the total sample (*n* = 113).

^c^ Restricted to the individuals who answered ‘NO’ regarding the use of N_2_O/O_2_ of the total sample (*n* = 113).

^d^ Correct answer.

^e^ Restricted to the individuals who do not attend a course (*n* = 79) but expressed the willingness to do it.

^f^ Restricted to the participants who answered ‘Agree’ on a 3‐point Likert scale (*n* = 113).

^g^ More than one answer option was available for participants.

### 5.3 Use and Inclination to Use Nitrous Oxide/Oxygen in Clinical Practice

A total of 40.7% (*n* = 46) of participants reported using N_2_O/O_2_ in their clinical unit to reduce children's anxiety and pain.

Regarding the frequency of N_2_O/O_2_ use in clinical practice, 59.3% (*n* = 67) of participants reported that they had never used it. Among non‐users (*n* = 67), 91% (*n* = 61) expressed a willingness to use it.

### 5.4 Knowledge of Guidelines, Laws, Requirements and Perceived Risks

While 68.1% (*n* = 77) of participants were unaware of existing local guidelines or regulations concerning the use of N_2_O/O_2_ sedation in paediatric patients, 31.9% (*n* = 36) reported being aware of them. Moreover, 57.5% (*n* = 65) of the study sample reported being aware that this type of sedation can be administered autonomously by a nurse if properly trained after a physician's prescription.

Regarding perceived risks associated with the use of N_2_O/O_2_, 33.6% (*n* = 38) of respondents identified dysphoria, vomiting, nausea and transient dizziness as potential side effects, whereas 31% (*n* = 35) reported no side effects.

### 5.5 Training and Provision of Education on Nitrous Oxide/Oxygen Use

Only 30.1% (*n* = 34) of participants reported having received specific training on N_2_O/O_2_ during their careers, while the majority (69.9%, *n* = 79) had not. Among these, 94.9% (*n* = 75) expressed their willingness to attend a dedicated training course on this topic.

### 5.6 Ethical and Organisational Perspectives on Nitrous Oxide/Oxygen Use

Most respondents, 91.1% (*n* = 103) considered the use of N_2_O/O_2_ to be ethically acceptable, aligning with the principles of beneficence, non‐maleficence and respect for child comfort. A total of 61.1% (*n* = 69) agreed that the procedure is cost‐effective, while the remaining 38.9% (*n* = 44) adopted a neutral stance and none disagreed.

22.1% (*n* = 25) of respondents answered that the use of N_2_O/O_2_ was perceived to slow down clinical workflow. When asked about the main barriers to the widespread adoption of N_2_O/O_2_ in paediatric care, understood as general practice rather than specific conditions within the study institutions, the most frequently reported issues included organisational concerns: the lack of appropriate facilities or equipment (92.9%, *n* = 105) and insufficient professional training (84.1%, *n* = 95).

Overall, 70.8% (*n* = 80) of respondents considered the use of N_2_O/O_2_ during non‐invasive paediatric procedures (e.g., wound dressing, venous access placement and suturing) to be safe, while the remaining 29.2% (*n* = 33) adopted a neutral stance and none disagreed.

Only 7.9% (*n* = 9) of participants thought that parents were aware of the option of conscious sedation with N_2_O/O_2_, but the majority (87.6%, *n* = 99) stated that, once adequately informed, parents would likely consent to its use.

### 5.7 Predictors of Nitrous Oxide/Oxygen Use: Multivariate Logistic Regression Results

A multivariate logistic regression model was conducted to identify independent predictors of N_2_O/O_2_ use among participants (Table 1).

The results indicate that department and educational level are significantly associated with the likelihood of using N_2_O/O_2_ in clinical practice. Specifically, professionals employed in maternal and child health units are markedly less likely to use N_2_O/O_2_ than those working in emergency departments (OR = 0.03; 95% CI: 0.01–0.16; *p* < 0.001).

Regarding educational level, respondents holding a master's degree in nursing or higher exhibit lower odds of using N_2_O/O_2_ than those with a three‐year nursing diploma (OR = 0.18; 95% CI: 0.05–0.68; *p* = 0.011).

## 6 Discussion

### 6.1 Knowledge, Training and Organisational Barriers

The aim of this study was to investigate the clinical knowledge, use and perspectives of N_2_O/O_2_ among physicians and nurses working in paediatric and emergency departments in Italy.

Only 29.2% of participants correctly answered questions on N_2_O/O_2_'s side effects. This finding is similar to those of Riccò et al. (2023), who found that Italian physicians had a ‘largely unsatisfactory’ understanding of N_2_O/O_2_ abuse, with an average knowledge score of 45.33% (±24.71) among those who reported prior familiarity with N_2_O/O_2_ (*n* = 115) [23].

Limited staff training appears to be a key barrier to the effective use of procedural sedation and analgesia (PSA) in Italian paediatric emergency care [24]. Sahyoun et al. (2021) [25] highlighted limited staff training in paediatric advanced life support and procedural sedation as a critical gap in emergency care, with only one‐third of emergency departments reporting full certification among clinicians performing paediatric PSA.

The Italian Consensus Conference (2017) [26] similarly underscored the need for dedicated training programs to ensure the safe administration of PSA and to support paediatricians and emergency physicians with appropriate skills.

Recent simulation‐based training initiatives during the COVID‐19 pandemic further confirmed the importance of structured education in ensuring safe N_2_O/O_2_ sedation practices.

These approaches enabled providers to practice independently, enhance their skills and increase their confidence in administering N_2_O/O_2_ sedation, with reported confidence increasing from 18% to 77% among 22 participants who completed the video‐based curriculum [27].

Nonetheless, data show that only 30.1% (*n* = 34) of healthcare providers had attended a specific training course on the use of N_2_O/O_2_ during their careers.

Furthermore, paediatric emergency medicine (PEM) is not formally recognised as a subspecialty [24], which may lead to the absence of specific national standards or curricular recommendations to ensure adequate PSA training for paediatricians or emergency physicians [26].

Accordingly, in this study, most participants were unaware of existing guidelines on N_2_O/O_2_ use, despite local guidelines being available in the reference hospitals. This aligns with Bevacqua et al. (2023) [24], who found that safety and monitoring guidelines for PSA were implemented in only half of Italian sites, and with Sahyoun et al. (2021), who reported similarly limited guideline implementation across European emergency departments [25].

Regarding barriers, 92.9% of respondents indicated that the lack of appropriate facilities or equipment was a significant obstacle to the N_2_O/O_2_ use. Limited availability of equipment could be explained by previous studies regarding organisational and infrastructural limitations. Particularly, the lack of physical space was commonly identified as a barrier to the implementation of PSA [1, 2]. Aligned with these findings, a Swiss study identified limited dedicated space (78%) and staffing shortages (89%) in hospital settings as major obstacles [28]. Similarly, in Canada, the most reported barriers to the use of N_2_O/O_2_ were concerns about ventilation and scavenging systems (71.2% of 80 physicians), as well as limited familiarity with the equipment (52.5%) [5]. Therefore, equipment availability could be linked to the need for adequate physical environments, infrastructure and trained personnel, without which, safe implementation cannot be achieved. The lack of equipment may indirectly compromise the quality of patient care [29], exposing patients to adverse events that increase morbidity and disability, leading to economic burdens on healthcare systems [30].

Despite existing limitations, evidence indicates a broad willingness to adopt N_2_O/O_2_, with 91.0% of non‐users expressing interest in its implementation, highlighting the need for greater investment in structural and organisational resources within the Italian National Health Service. The lack of investment in appropriate sedation equipment may exacerbate inequalities in care and expose patients to avoidable risks. Its underuse may not only depend on infrastructural barriers but also, sometimes, on ethical concerns in balancing patient comfort with clinical goals [31]. This must be supported by national guidelines, ongoing professional training, anaesthesiologic support and suitable clinical environments.

### 6.2 Utilisation Rates and International Trends

In this study, only 40.7% of Italian healthcare professionals currently use N_2_O/O_2_ in clinical practice, while 59.3% have never used it. Similarly, in Canada, 51.3% of physicians reported not using N_2_O/O_2_; among them, 93.7% cited its unavailability at their facility, yet most expressed a desire to access it [32]. Availability remains inconsistent in Canada as well, with only 40% of paediatric emergency departments reporting access to N_2_O/O_2_ [33], mirroring the situation observed in Italy.

### 6.3 Willingness to Use Nitrous Oxide/Oxygen

When available, N_2_O/O_2_ is valued for its effectiveness and safety, particularly in procedures such as digit fractures or dislocations, wound suturing, incision and drainage [33]. A favourable safety profile for N_2_O/O_2_ has been reported across studies. A recent systematic review found that N_2_O/O_2_ used in paediatric emergency procedures was generally well tolerated, with adverse events mostly mild and transient [32]. Similarly, for painful orthopaedic procedures in children, the combination of intranasal fentanyl and inhaled N_2_O/O_2_ is as effective as intravenous ketamine and midazolam, with a significantly better safety profile and shorter emergency department stay [33].

Despite this, safety concerns were also raised in dental settings, particularly regarding the increased risk of foreign body aspiration during conscious sedation with N_2_O/O_2_ [34], where the gag reflex may be suppressed. Additionally, concerns remain among families; in a survey of paediatric dentists, 51% reported parental reservations about using N_2_O/O_2_ on their children, primarily regarding safety, sedation depth, systemic duration and potential neurological effects [35], highlighting the need for transparent communication. Notably, 87.6% of professionals indicated that, once properly informed, parents were likely to consent to N_2_O/O_2_ administration.

### 6.4 Predictors of Nitrous Oxide/Oxygen Use in Paediatric Clinical Settings

The multivariate analysis identified two main predictors of N_2_O/O_2_ use in paediatric practice: healthcare professionals' educational level and clinical setting.

Professionals holding a 3‐year degree or a diploma tended to use N_2_O/O_2_ more frequently than those with higher academic qualifications. Although this finding may appear counterintuitive, the study did not collect data on specific training received; therefore, this factor should be considered when interpreting the results. Further studies are warranted to explore this issue more in depth.

The clinical setting also emerged as a relevant factor influencing the likelihood of N_2_O/O_2_ use. In this study, N_2_O/O_2_ use was reported by 44 clinicians from emergency units (59.5% of 74 respondents), compared with 2 clinicians from maternal–infant units (5.1% of 39 respondents). However, when interpreting the results, it is important to consider that participants employed in maternal units were underrepresented compared to those in emergency units. This difference cannot be directly explained by the existing evidence; however, a Canadian survey of paediatric emergency physicians reported that equipment availability and prior clinical experience with N_2_O/O_2_ were the most frequently perceived facilitators of its use [33]. Suggesting that differences between units may be due to equipment availability, which also affects clinicians' experience with the gas.

Additionally, the higher use of N_2_O/O_2_ by emergency professionals may be explained by their greater familiarity with its use in urgent settings. Indeed, its administration requires no fasting period and can be easily self‐administered [6], making it particularly suitable for emergency care. These features likely contribute to the higher engagement of emergency clinicians compared with those from maternal–infant units, as shown by data in this study.

Although structural barriers, such as limited equipment and training, remain the main obstacles to N_2_O/O_2_ implementation, demographic predictors are also clinically relevant. Indeed, identifying which groups are more likely to use N_2_O/O_2_ can guide targeted interventions, allow policymakers and educators to prioritise resource allocation, develop tailored training programs, maximising the impact of infrastructural enhancements.

## 7 Limitations

Despite efforts to maintain adequate methodological rigour throughout the study, several limitations should be acknowledged.

First, the cross‐sectional design, based on data collected at a single point in time, precludes the establishment of causal relationships between variables. Non‐probabilistic sampling and self‐reported data may limit generalizability due to selection and social desirability biases. Furthermore, conclusions on the maternal–infant setting should be interpreted with caution, as the number of professionals involved was considerably lower compared to the emergency setting.

Another limitation may be not to have considered whether the training received on N_2_O/O_2_ could be considered a predictive factor for its use and for the identification of side effects and whether awareness of the guidelines could have affected the identification of side effects by the participants.

## 8 Implications and Recommendations for Practice and Further Research

This cross‐sectional study provides an assessment of the knowledge, clinical use and perspective of N_2_O/O_2_ in paediatric healthcare settings in Italy. The data clearly show that a significant proportion of healthcare staff are in favour of using N_2_O/O_2_, despite never having applied it directly. Clear guidelines, checklists and standardised protocols are needed to support consistent and safe use. Additionally, training programs should support both technical skills and ethical confidence in N_2_O/O_2_ use.

Finally, further research should be conducted to validate these findings assessing the long‐term impact of such interventions on clinical outcomes and care quality, especially on the maternal–infant setting to better understand the differences observed across clinical contexts.

## 9 Conclusion

This study aimed to explore the actual knowledge, use and perspectives of Italian healthcare professionals regarding N_2_O/O_2_ in paediatric and emergency units. Participants recognised its clinical, ethical and cost‐effective value. However, persistent barriers were identified, including a lack of standardised protocols, insufficient equipment and limited access to accredited training, particularly in non‐emergency settings. Raising awareness among healthcare managers and policymakers on the clinical, ethical and economic value of safe sedation practices is crucial to support the wider implementation of N_2_O/O_2_.

Predictors, such as setting and educational level, could inform future strategies where targeted training and broader protocol implementation are key to improving N_2_O/O_2_.

## Author Contributions

V.S., G.C. responsible for the conception and design of the work, acquisition and interpretation of data; D.C., B.G. contributed to the conception and the design of the work, acquisition and interpretation of data; L.M. responsible for the statistical analysis, methodology, formal analysis, validation, writing – original draft; D.M., I.S., E.C., S.O., M.A. contributed to the conception of the work and to the acquisition of data; V.S., G.C., D.C., B.G. responsible for writing – original draft – revised critically the word for important intellectual content. All authors approved the final version to be published; all authors agreed to be accountable for all aspects of the work in ensuring that questions related to the accuracy or integrity of any part of the work are appropriately investigated and resolved.

## Funding

This research was supported by Universita degli Studi Gabriele d'Annunzio Chieti Pescara, as part of the Wiley – CRUI‐CARE agreement.

## Ethics Statement

Ethical approval was granted by the Territorial Ethical Committee of the Marche Region (CET M) on February 15, 2024, protocol number 2024 8.

## Consent

Written informed consent was obtained from study participants.

## Conflicts of Interest

The authors declare no conflicts of interest.

## Data Availability

The data presented in this study are available on request from the corresponding author.
